# The Early Dead

**Published:** 1857-02

**Authors:** 


### THE EARLY DEAD.

In describing "Old Princeton," after an absence of eighteen years, the Rev. Dr. Hill, the able editor of the *Presbyterian Herald*, of Kentucky, says: "Of the nearly one hundred and fifty students who then trod the halls of the seminary, a greater number have rested from their labors."

There was a time when, of all the clergymen dying in a single year whose ages were recorded, more than one-half had passed the age of three-score and ten. But of the cœvals of Dr. Hill's seminary life, more than one-half scarcely passed their fortieth year. Here is a cutting short of clerical life of a quarter of a century! Ought not a fact of this kind elicit inquiry among the friends of religion everywhere? And is there not an inconsistency on the part of the Church, in so earnestly soliciting the contributions of the public for money to aid young men to enter the ministry, and yet not devote a single dollar in an age towards the means of keeping them in the field until a good old age, by instructing them as to the best means of sustaining their health in full vigor? A good general is careful of the health of his soldiers. Common policy requires it, not only because the veteran of a dozen battles is worth more than a dozen young recruits, but because of the cost in putting a soldier in the field of his operations. It costs at least two thousand dollars to take a youth of eighteen and put him into the full ministry; and yet, as to the preservation of his health, and as to taking measures to keep him in these until he grows old in the service, no system has ever yet been proposed—not a dollar has hitherto been specifically expended.

